# An odorant-binding protein in the elephant's trunk is finely tuned to sex pheromone (*Z*)-7-dodecenyl acetate

**DOI:** 10.1038/s41598-022-24214-5

**Published:** 2022-11-21

**Authors:** Valeriia Zaremska, Giovanni Renzone, Simona Arena, Valentina Ciaravolo, Andreas Buberl, Folko Balfanz, Andrea Scaloni, Wolfgang Knoll, Paolo Pelosi

**Affiliations:** 1 Austrian Institute of Technology GmbH, Biosensor Technologies, Konrad-Lorenz Straße, 24, 3430 Tulln, Austria; 2 Proteomics, Metabolomics and Mass Spectrometry Laboratory, ISPAAM, National Research Council, 80055 Portici, Italy; 3 Vienna Zoo, Maxingstraße 13B, Vienna, Austria; 4 Department of Physics and Chemistry of Materials, Faculty of Medicine/Dental Medicine, Danube Private University, Krems, Austria

**Keywords:** Ecology, Zoology, Biochemistry, Proteins, Proteomics

## Abstract

Chemical communication in elephants has been well studied at the chemical and behavioural levels. Pheromones have been identified in the Asian elephant (*Elephas maximus*), including (*Z*)-7-dodecenyl acetate and frontalin, and their specific effects on the sexual behaviour of elephants have been accurately documented. In contrast, our knowledge on the proteins mediating detection of pheromones in elephants remains poor and superficial, with only three annotated and reliable entries in sequence databases, two of them being odorant-binding proteins (OBPs), and the third a member of von Ebner's gland (VEG) proteins. Proteomic analysis of trunk wash extract from African elephant (*Loxodonta africana*) identified one of the OBPs (LafrOBP1) as the main component. We therefore expressed LafrOBP1 and its Asian elephant orthologue in yeast *Pichia pastoris* and found that both recombinant proteins, as well as the natural LafrOBP1 are tuned to (*Z*)-7-dodecenyl acetate, but have no affinity for frontalin. Both the natural and recombinant LafrOBP1 carry post-translational modifications such as O-glycosylation, phosphorylation and acetylation, but as these modifications affect only a very small amount of the protein, we cannot establish their potential effects on the ligand-binding properties of OBP1.

## Introduction

The order Proboscidea is phylogenetically separated from all other mammals and includes three living species, the Asian elephant (*Elephas maximus*) and the two African species, the savanna and the forest elephant (*Loxodonta africana* and *L. cyclotis*, respectively)^1^, besides the extinct mammoths. In this work, we focus on the savanna African elephant *L. africana* as no chemistry on chemical signalling by the forest African elephant *L. cyclotis* has been published to our knowledge.

Communication between sexes in elephants has been mainly studied at the chemical and behavioural level, thanks to the long and accurate work of the late Elizabeth Rasmussen and her collaborators, who continued her research producing several interesting and unique results^2^. Two sex pheromones were first identified in the Asian elephant: (*Z*)-7-dodecenyl acetate, which is released in the urine of pre-ovulatory females, and frontalin {1,5-dimethyl-6,8-dioxabicyclo[3.2.1]octane}, the main component of the male temporal gland secretion (TGS) during musth^3–8^. Intriguingly, both compounds are also pheromones for a large number of Lepidoptera and Coleoptera, respectively^9^. Later work reported the presence of the same compounds in the urine and in the TGS of female African elephants, together with a number of other volatiles, such as *exo*- and *endo*-brevicomin {(1R,5S,7R)-7-ethyl-5-methyl-6,8-dioxabicyclo[3.2.1]octane and (1R,5S,7S)-7-ethyl-5-methyl-6,8-dioxabicyclo[3.2.1]octane, respectively }, (*E*)-β-farnesene [(6E)-7,11-dimethyl-3-methylene-1,6,10-dodecatriene] and (*E*,*E*)-α-farnesene [(3E,6E)-3,7,11-trimethyldodeca-1,3,6,10-tetraene], all known also as insect pheromones^9^. Moreover, frontalin was also detected in the blood of musth males^10^. An interesting aspect is the role of chirality played in the action of frontalin. In young males, the ( +)-enantiomer is predominant in TGS, but the presence of its optical antipode increases with age reaching the composition of a racemate at full maturity^11^. On the contrary, females produce almost pure (−)-frontalin^10^. Also both enantiomeric forms of *endo*- and *exo*-brevicomin occur in blends of the four stereoisomers in variable ratios^9,10^.

Behaviour has been extensively investigated, also using the identified pheromones, and generated a wealth of interesting and novel information^6,10,12^. This can briefly summarised as follows:

1. Males are attracted to the urine of females in estrus and communicate their readiness with frontalin secreted by TGS^13^. While musth is observed regularly in males, estrous in females occurs only every 4–5 years, after a two-year gestation and a long weaning period.
2. Frontalin produces different responses in males and females depending on age and physiological condition. At the beginning, male elephants produce a TGS containing floral smelling compounds, but no frontalin, which is only synthesised with the onset of maturity and increases its levels many folds as the animal gets older^11,14^.
3. Frontalin does not produce significant behaviour in old males nor in females in the luteal phase. Instead, young males and pregnant females get apprehensive and try to avoid this pheromone. On the contrary, females in the follicular phase show attraction to this chemical^8^.
4. Elephants can discriminate between the two enantiomers of frontalin. It has also been observed that females respond much stronger to racemic frontalin than to different mixtures of the two enantiomers. In fact, the racemate is a sign of maturity, whereas the (+)-enantiomer predominates in young males, and the (−)-enantiomer is the only one produced by females^10,12^

In this work, we have investigated the role of soluble odorant-binding proteins (OBPs) in sexual chemical communication of two elephant species, the Asian and the African elephants, and we also have measured their affinities to the pheromone components. In mammals, such proteins mediate chemical communication between sexes by a dual mechanism; in the nose, they carry pheromones to membrane-bound receptors, while in specialised glands and secretions, they solubilise the same pheromones for their delivery in the environment^15^. OBPs are small soluble polypeptides present in large amounts in the nasal mucus^16–18^, as well as in body fluids such as urine, saliva, seminal fluid and other external secretions^19,20^. They bind species-specific pheromones with dissociation constants in the upper nanomolar/lower micromolar range. Although tuned to their target pheromones, their specificity is rather broad, exhibiting weaker binding activity to some structurally related compounds. Recent work has shown that post-translational modifications (PTMs), such as glycosylation and phosphorylation play important roles in tuning the specificity of OBPs to their target ligands by increasing affinity, narrowing specificity and modulating the binding spectrum^21^.

An OBP was detected in the trunk wash of the Asian elephant and shown to bind (*E*)-7-dodecenyl acetate^22^. The adopted assay involved separation of a crude protein extract of the trunk wash on an electrophoretic gel after incubation with tritium-labelled (*E*)-7-dodecenyl acetate. Radioactivity associated with the OBP band indicated specific binding, but did not allow evaluation of the dissociation constant.

Here, we report on the proteomic analysis of the trunk wash from the African elephant, the expression of two orthologue OBPs from the Asian and the African elephants in the yeast *Pichia pastoris*, and the characterization of their binding activity.

## Results and discussion

### Sequence analysis

**Figure 1:**
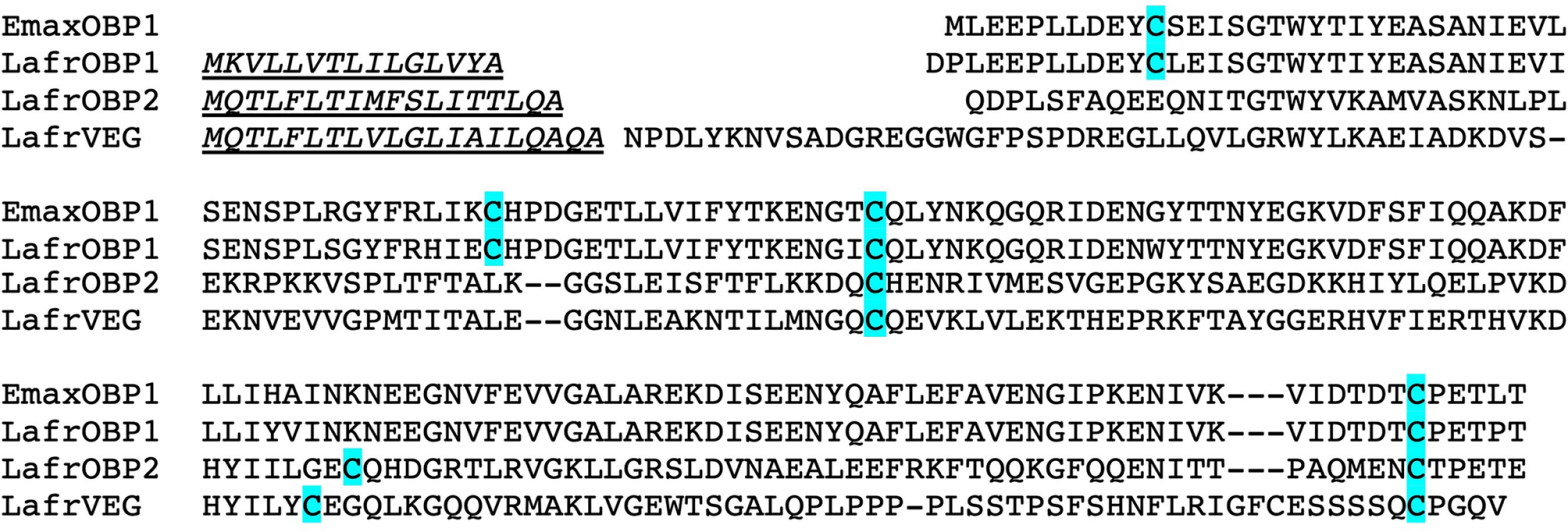
Two orthologous sequences in the Asian elephant *Elephas maximus* (EmaxOBP1: AAN17333) and in the African elephant *Loxodonta africana* (LafrOBP1: XP_023395442) share 93% of their residues and are annotated as OBP1. Another sequence, found only in the African elephant (LafrOBP2: SP_tr|G3U771) is only 15% identical with LafrOBP1. In addition, a member of the VEG proteins, found only in the databases of the African elephant (LafrVEG: XP_023398512.1), is 13% and 31% identical with LafrOBP1 and LafrOBP2, respectively. Predicted signal peptides are indicated in italics and underlined. The sequence of EmaxOBP1 is found in the databases as reported, without a signal peptide. Cysteine residues are highlighted.

The OBPs of elephants are not completely annotated. A search in different sequence databases (NCBI and UniProtKB) returned two orthologous sequences for the African and the Asian elephant, sharing 93% of their residues, which we here indicate as LafrOBP1 and EmaxOBP1 (Fig. 1). In the African elephant, two additional sequences are annotated. The first, which here we have named LafrOBP2, presents only 15% identity with LafrOBP1. The second (LafrVEG), 13% identical to LafrOBP1, bears a significant similarity (30–40% identity) to a group of lipocalins known as VEG proteins, tear prealbumins or lipocalin-1 (LCN1). A function of the latter polypeptides as carriers for taste compounds had been suggested at the beginning^23^ but this idea was not strongly supported by experimental evidence and alternative roles were proposed, such as scavenger for toxic chemicals and transporter for vitamins and nutrients^24^, without however disproving the original hypothesis. The sequences of these four lipocalins are aligned in Fig. 1. In addition, some isoforms of both LafrOBP1 and LafrOBP2 were found in the databases, but their sequences were not fully reliable, being incomplete or too long at their C-terminus.

### Proteomic analysis of whole trunk wash

We first decided to analyse a sample of the trunk wash from the African elephant to identify the proteins secreted, with particular attention to OBPs. Therefore, the sample was filtered, concentrated and separated on an electrophoretic gel in denaturing conditions (SDS-PAGE) (Figure S1). Abundant bands were observed in the low molecular weight region (20–25 kDa), which represented most of the proteins in the trunk wash. Slices were cut from the gel, reduced, alkylated with iodoacetamide, digested with trypsin and subjected to nanoLC-ESI-Q-Orbitrap-MS/MS analysis. We identified three abundant lipocalins corresponding to the sequences XP_023395442.1, XP_023395433.1 and XP_023398512.1. The first one coincides with LafrOBP1 annotated in the database, the second is an isoform of LafrOBP1 bearing the amino acid substitutions Leu13Ser, Ile32Leu, Glu78Lys, Trp80Gly, Glu116Lys, and additional sequence portions at both N- and C-terminal region while the third one corresponds to LafrVEG mentioned above. We could not detect the presence of LafrOBP2. A summary of the results of this proteomic analysis is reported in supplementary Table S1.

### Fractionation of soluble proteins from trunk wash and analysis of PTMs in LafrOBP1

**Figure 2:**
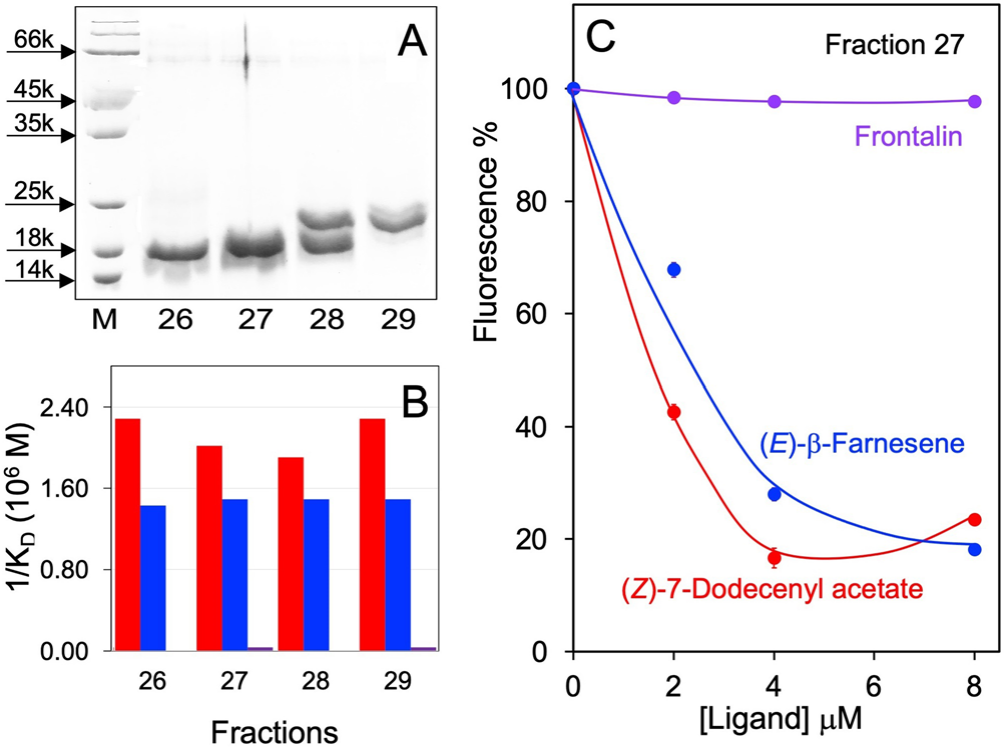
(**A**) Fractionation of a crude extract of trunk wash from the African elephant by anion-exchange chromatography. SDS-PAGE analysis of the fractions showing electrophoretic bands in the low molecular weight range (15–25 kDa). (**B**) Summary of the affinities to (*Z*)-7-dodecenyl acetate, (*E*)-β-farnesene and frontalin measured with crude samples of the same fractions. All of them bound with good affinity both (*Z*)-7-dodecenyl acetate and (*E*)-β-farnesene. Also farnesol, proved to be as good a ligand as (*E*)-β-farnesene, whereas *exo*-brevicomin did not show any affinity. Dissociation constants were evaluated using the procedure adopted when dealing with solutions of a pure protein; as this is not the case, the values have to be taken as a crude indication of the affinity of the ligands for the protein. (**C**) Examples of the competition curves measured with the three ligands on fraction 27.

Given the predominant presence of LafrOBP1 in the trunk wash of the African elephant, we attempted purification of this protein from a crude sample of this secretion by anion-exchange chromatography. Relevant fractions (#26–29) were analysed by SDS-PAGE, (Fig. 2A) and nanoLC-ESI-Q-Orbitrap-MS/MS to identify the proteins present and detect possible PTMs. The same fractions were also tested in ligand-binding assays with some of the two elephants' pheromones and other potential semiochemicals to detect any binding activity (Fig. 2B-C). All fractions bound (*Z*)-7-dodecenyl acetate with low dissociation constants (0.44–0.52 μM) and showed a good affinity to (*E*)-β-farnesene (K_D_: 0.67–0.70 μM). On the contrary, none of them appreciably bound frontalin. We also tested farnesol, which proved to be as good a ligand as (*E*)- β-farnesene, and *exo*-brevicomin, which did not exhibit any affinity (data not shown).

**Figure 3:**
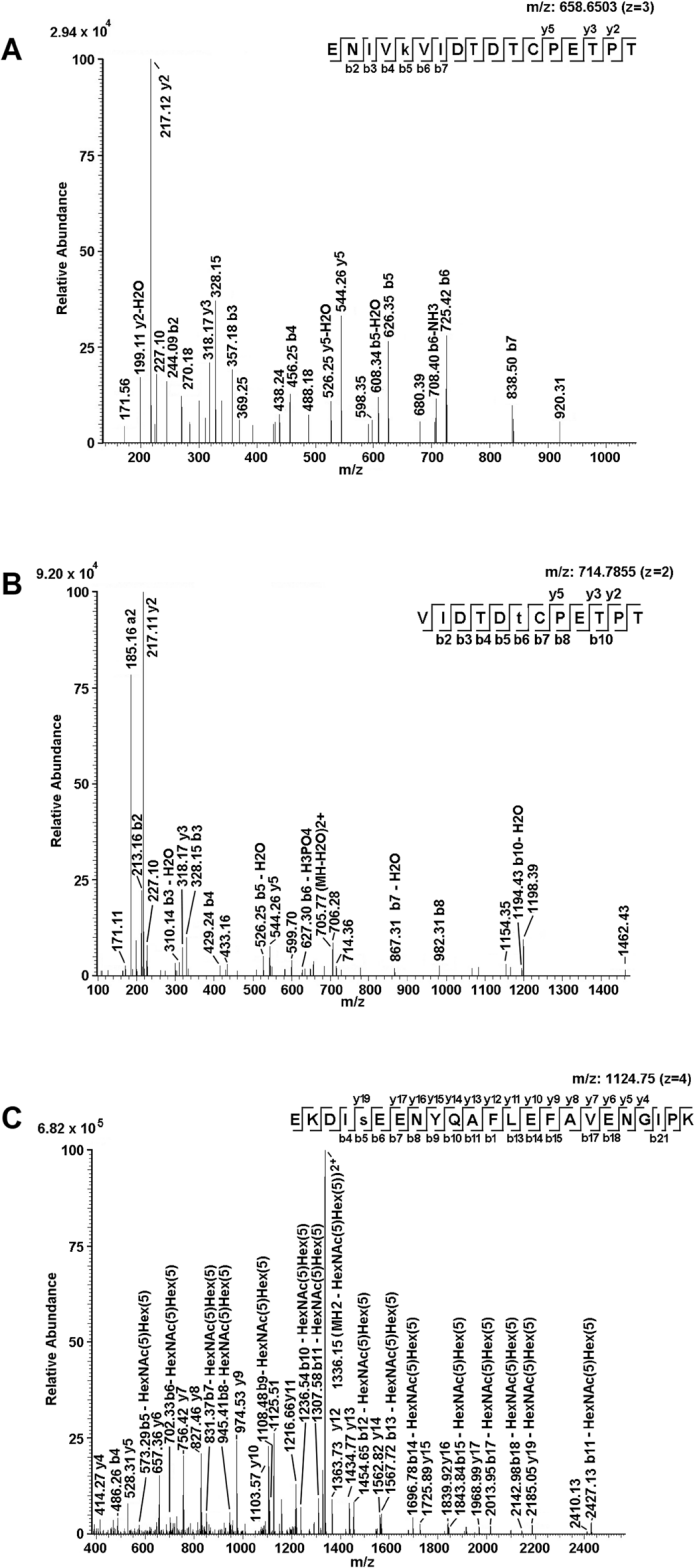
Examples of post-translationally modified peptides assigned to LafrOBP1 from trunk wash of the African elephant. An acetylated peptide, a phosphorylated peptide and the O-glycosylated peptide are shown. Identical peptides and corresponding fragmentation spectra were observed also in the recombinant protein expressed in the yeast *Pichia pastoris* (see Table S2). Panel A shows the fragmentation spectrum of the acetylated peptide (147–163) bearing modification at Lys151. Panel B shows the fragmentation spectrum of the phosphorylated peptide (152–163) bearing modification at Thr157. Panel C shows the fragmentation spectrum of the O-glycosylated peptide (124–146) bearing a tetra-*N*-acetyl-lactosamine-containing core-2 O-glycan linked to Ser128.

Proteomic analysis of fractions 26–29 demonstrated the concomitant occurrence in each fraction of LafrOBP1, its isoform and LafrVEG, highlighting the difficulty to resolve these proteins based on anion-exchange chromatography. An independent bioinformatic search of mass spectrometric data including various putative PTMs allowed identifying modified peptides from native LafrOBP1, including acetylated, phosphorylated and O-glycosylated ones (Table S2). Abundant non-modified corresponding peptides were also identified in each fraction. Exemplificative tandem mass spectra of modified peptides are reported in Fig. 3. As expected, slight differences were observed in the representation of modified peptides in the different chromatographic fractions (Table S2). In the whole, acetylated peptides (60–71), (62–75), (76–98), (88–98), (124–146), (124–151) and (147–163), and phosphorylated components (44–61) and (152–163) were observed in the digests of native LafrOBP1*.* Fragmentation spectra of above-mentioned species definitively assigned acetylation at Lys61, Lys71, Lys88, Lys125, Lys146 and Lys151, as well as phosphorylation at Tyr59 and Thr157 (Table S2 and Figure S2). Fractions 27–29 also showed the occurrence of the O-glycosylated (124–146) peptide bearing a tetra-*N*-acetyl-lactosamine-containing core-2 O-glycan^25^ (Table S2), which was linked to Ser128, according to its tandem mass spectrum (Fig. 3).

### Expression and purification of LafrOBP1 and EmaxOBP1

Based on above-reported proteomic analysis and on the available sequences in the protein databases, we decided to focus our functional study on the OBP1 of both species. Thus, LafrOBP1 and EmaxOBP1 were expressed in the yeast *Pichia pastoris* using the protocol reported in the Materials and Methods section. We chose to perform functional studies on recombinant proteins because attempts to purify natural LafrOBP1 from its contaminants were unsuccessful (data not shown). The recombinant proteins were secreted in the culture medium, concentrated by ultrafiltration and purified by anion-exchange chromatography, followed by a step of hydrophobic interaction chromatography on Phenyl Sepharose. Figure S3 reports the SDS-PAGE analysis relative to the expression and purification of the two proteins. The expression of EmaxOBP1 produced a single major band migrating with an apparent molecular weight (around 20 kDa), compatible with the calculated mass for this protein (18,506.7 Da). Instead, the expression of the African elephant orthologue afforded two major bands, the first migrating at the same level as EmaxOBP1, the second with an apparent mass around 25 kDa. This second band was also purified, but, unlike the lower band, did not show any affinity to the fluorescent probe N-phenyl-1-naphthylamine (1-NPN), therefore, it was not considered further in our study.

### Structure analysis and PTMs of recombinant LafrOBP1

To identify PTMs in the recombinant LafrOBP1 and compare them with those detected in the natural protein, the first was also subjected to proteomic analysis. We found that most of the modifications identified in the natural LafrOBP1 were conserved in the recombinant product. In fact, acetylated species (60–71), (62–75), (124–146) and (124–151), phosphorylated peptides (44–61), (62–75) and (152–163), as well as the O-glycosylated (124–146) component bearing the tetra-*N*-acetyl-lactosamine-containing core-2 were also detected in the digest of recombinant LafrOBP1 (Table S2 and Figure S2). Abundant non-modified corresponding peptides were also found in recombinant LafrOBP1. Fragmentation spectra of above-mentioned modified peptides further confirmed acetylation at Lys61, Lys71, Lys125 and Lys146, phosphorylation at Tyr59, Tyr69 and Thr157, and O-glycosylation at Ser128 (Table S2 and Figure S2). The slight modification differences observed with respect to the natural protein (Figure S2) were rationalized based on the distinct modification machineries present in the African elephant and *P. pastoris*.

**Figure 4:**
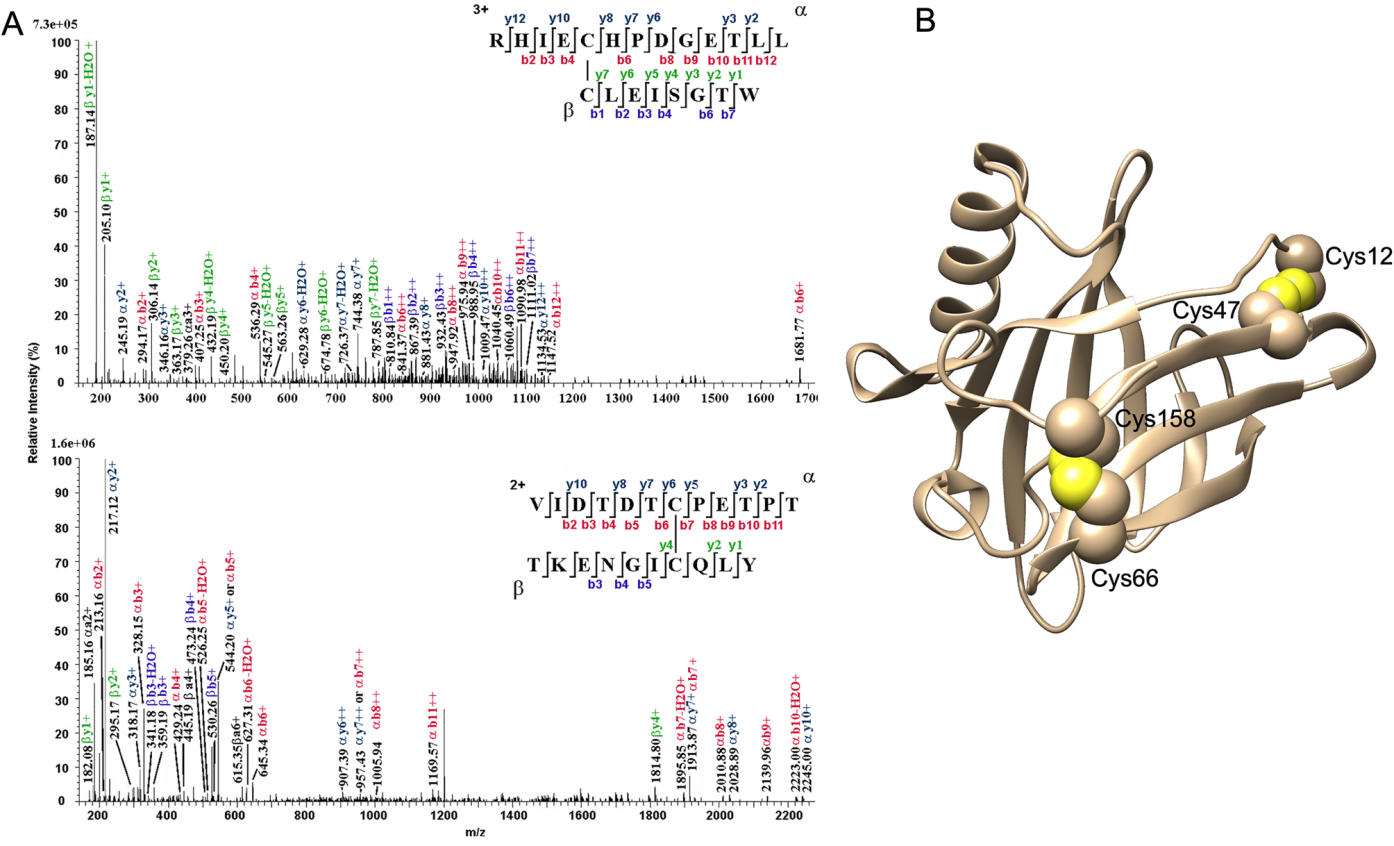
(**A**) Fragmentation spectra of disulfide-bridged peptides identified in the tryptic-chymotryptic digest of recombinant LafrOBP1. The upper and the lower panels show independent data relative to Cys12-Cys47 and Cys66-Cys158 disulfide bridges. The fragment ions are reported in different color depending on the peptide present in S–S-linked species, and corresponding b and y ion series. (**B**)Three-dimensional model of LafrOBP1 showing the pairing of the four cysteines. The model was generated with the on-line software Swiss Model using the structure of the allergen Bosd2 (PDB: 4WFU.1.A) as a template.

LafrOBP1 and EmaxOBP1 contain four cysteines occurring at conserved positions in two elephant species (Fig. 1), but differing in number and distribution from what is observed in other mammalian OBPs^26^. To ascertain their oxidation state and their potential pairing in disulfide bonds, recombinant LafrOBP1 was subjected to extensive alkylation with iodoacetamide under non-reducing denaturing conditions; then, it was digested first with trypsin and then by chymotrypsin. Resulting peptides were analyzed by nanoLC-ESI-Q-Orbitrap-MS/MS, and peptides linked by disulfide bridges were identified by dedicated bioinformatic procedures^27–29^. Detection of peptides (12–19)S–S(43–55) and (60–69)S–S(152–163) (Fig. 4A) demonstrated that all cysteines present in LafrOBP1 are involved in disulfide bridges in the pattern C1-C2 and C3-C4. This disulfide motif involves three conserved cysteines present in some homologous proteins from other mammals; the forth is peculiar to elephant OBP1.

A structural model of LafrOBP1 was generated using the on-line programme Swiss Model^30–32^ and the allergen Bosd2 (PDB: 4WFU.1.A) as a template, whose amino acid sequence is 31% identical to LafrOBP1. Apart from the good sequence similarity between the two proteins, the reliability of this model was also supported by the experimental evidence for the presence of the two disulfide bridges in LafrOBP1. Figure 4B reports the model of LafrOBP1 showing the four cysteines involved in disulfide bridges. As expected, various hydrophobic amino acids occurred in the protein binding pocket (data not shown), in agreement with what was observed in structurally related OBP 1F from the brown rat *Rattus norvegicus* (PDB: 3FIQ), aphrodisin from the golden hamster *Mesocricetus auratus* (PDB: 1E5P), allergen lipocalin Cav p1 from the guinea pig *Cavia porcellus* (PDB: 8A0D) and lipocalin allergen Bos d2 from the cattle *Bos taurus* (PDB: 1BJ7).

### Ligand-binding assays

**Figure 5:**
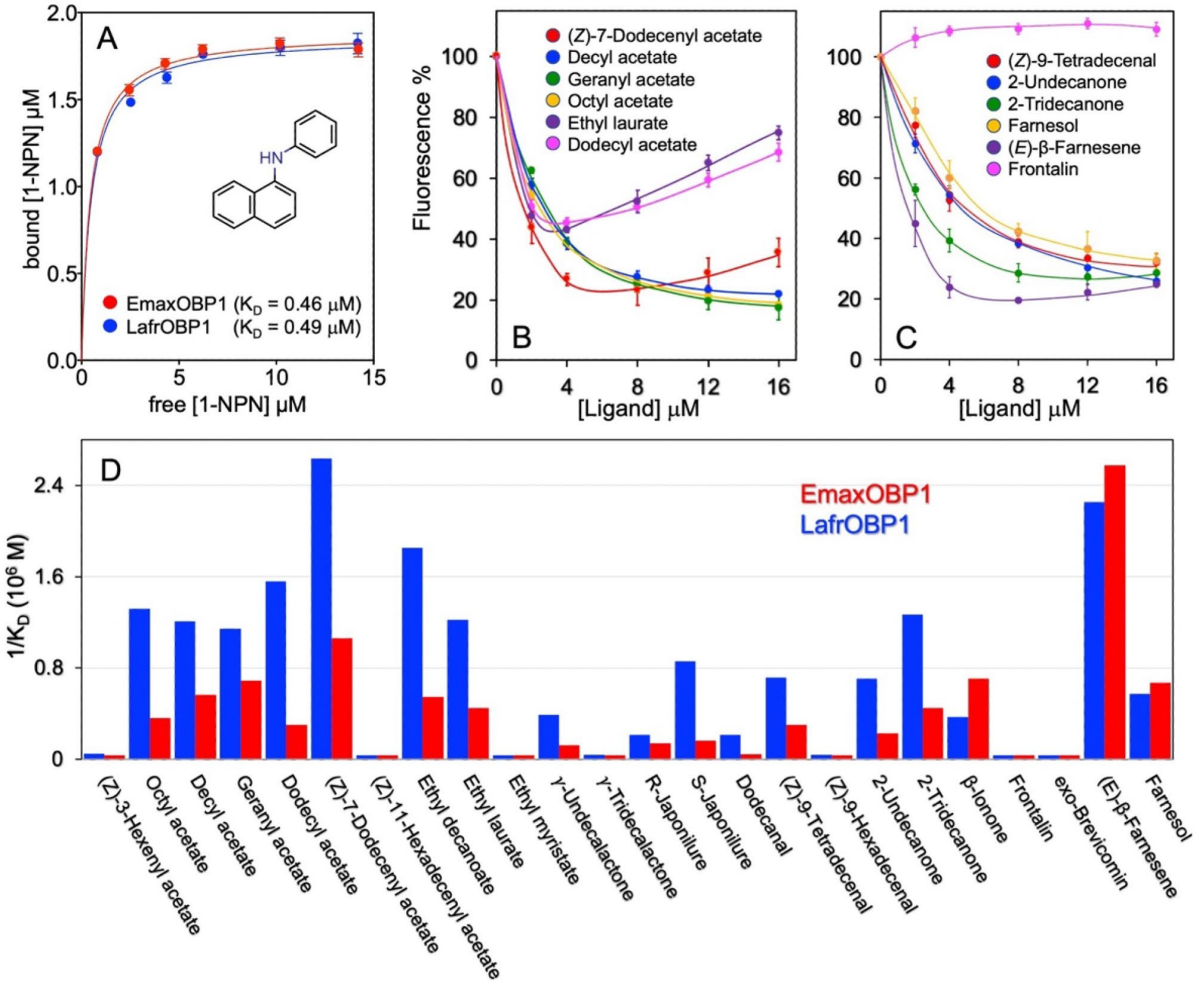
Binding of recombinant LafrOBP1 and EmaxOBP1 to the elephants' pheromones and structurally related compounds. (**A**) Both proteins bound the fluorescent probe N-phenyl-1-naphthylamine (1-NPN), whose structure is reported as an inset, with strong and very similar affinities. (**B**) Binding of the pheromone (*Z*)-7-dodecenyl acetate and few structurally related esters to recombinant LafrOBP1. The increase of the fluorescence at higher concentrations of ligand is due to formation of micelles by some amphiphilic ligands, which could entrap molecules of 1-NPN^33–35^. (**C**) Binding of representative compounds among the other tested chemicals. Interestingly, (*E*)-β-farnesene was found to be a good ligand, despite its structural difference with the Asian elephant pheromone (*Z*)-7-dodecenyl acetate. Instead, frontalin, a second Asian elephant pheromone, did not exhibit any affinity for the protein. (**D**) Graphical comparison of the affinities of selected ligands to the OBP1 of both elephant species. No major differences were observed in the binding spectra between LafrOBP1 and EmaxOBP1. In addition to the reported ligands, the following compounds did not exhibit significant affinity for neither of the two proteins: hexanal, octanal, decanal, (*Z*)-11-hexadecenal, (*Z*)-13-octadecenal, eucaliptol, *p*-cresol, eugenol.

**Figure 6:**
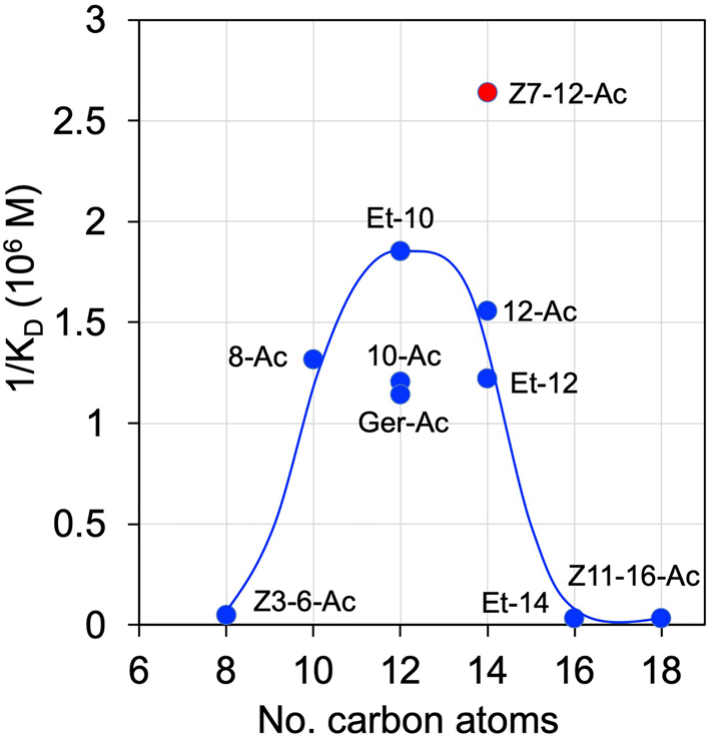
Affinities of some esters to LafrOBP1 plotted as a function of the number of carbon atoms. The pheromone (*Z*)-7-dodecenyl acetate was the strongest ligand, while some other esters of 10–14 carbon atoms also bound with lower affinities. Homologous esters of less than 10 or more than 14 carbon atoms did not show significant binding for LafrOBP1. Z3-6-Ac: (*Z*)-3-hexenyl acetate; 8-Ac: 1-octyl acetate; 10-Ac: 1-decyl acetate; Ger-Ac: geranyl acetate; Et-10: ethyl decanoate; Z7-12-Ac: (*Z*)-7-dodecenyl acetate; 12-Ac: 1-dodecyl acetate; Et-12: ethyl dodecanoate; Et-14: ethyl tetradecanoate; Z11-16-Ac: (*Z*)-11-hexadecenyl acetate.

Recombinant LafrOBP1 and EmaxOBP1 were then tested in ligand-binding assays using the Asian elephant pheromones (*Z*)-7-dodecenyl acetate and frontalin, and the insect pheromone components found in elephants, *exo*-brevicomin and (*E*)-β-farnesene, as well as structurally related compounds, and chemicals of different size and structure, which could contribute to estimate the specificity of these proteins. The results are reported in Fig. 5. Both LafrOBP1 and EmaxOBP1 bound the fluorescent probe 1-NPN with good affinity (Fig. 5A), thus enabling the use of the fluorescent displacement assay to evaluate the affinities of other ligands. Some representative curves of competitive binding experiments for LafrOBP1 are shown in Fig. 5B-C, while in the histogram of Fig. 5D the binding spectra of LafrOBP1 and EmaxOBP1 are compared. The specificity of LafrOBP1 can be better appreciated from the graph of Fig. 6, where the affinities of esters to the protein are plotted as a function of the ligand's size. In addition to the ligands reported in Fig. 5, the following chemicals did not show significant binding to either of the two proteins: (*Z*)-3-hexenyl acetate, (*Z*)-11-hexadecenyl acetate, ethyl myristate, γ-tridecalactone, octanal, decanal, dodecanal, (*Z*)-9-hexadecenal, (*Z*)-11-hexadecenal, (*Z*)-13-octadecenal, eucalyptol, borneol and eugenol.

To evaluate to what extent phosphorylation and glycosylation could account for the binding activity of LafrOBP1, fractions 27 and 29 from trunk wash and a sample of the protein expressed in *P. pastoris* were treated with Lambda phosphatase and O-glycosidase. In no case did we observe any shift in the apparent molecular weight of the protein. Figure S4A shows the results relative to treatment of the recombinant protein. We also found that after digestion with phosphatase the binding of (*Z*)-7-dodecenyl acetate to recombinant LafrOBP1 was not affected (Figure S4B).

In this work we have shown that:

1. Two OBPs, OBP1 and OBP2, are encoded in the genome of the elephants apart from VEG protein, whose functional similarity with OBPs has been suggested, but is not supported by strong evidence. In addition, some isoforms of both OBPs can be identified, but their sequences are not fully reliable.
2. The trunk wash of the African elephant contains OBP1, one of its isoforms and the VEG protein in significant concentrations.
3. The OBP1 isolated from the trunk wash of the African elephant occurs in several fractions separated by anion-exchange chromatography, all equally binding the Asian elephant sex pheromone (*Z*)-7-dodecenyl acetate, as well as β-farnesene and farnesol, but do not show any affinity to the musth pheromone frontalin or to *exo*-brevicomin.
4. Post-translational modifications, such as O-glycosylation, acetylation and phosphorylation, have been detected in both the protein extracted from the trunk wash and in the yeast-expressed LafrOBP1. However, as such modifications affect only a small fraction of the protein population, their effects on ligand-binding properties cannot be established.

## Materials and methods

### Materials

Trunk wash was collected from one male (Tembo, born 1985) and five female (Tonga, 1984; Numbi, 1992; Mongu, 2003; Iqhwa, 2013; Kibali, 2019) African elephants at the Vienna Zoo during routine procedures. Briefly, 100 mL of a sterile 0.9% saline solution is injected in each nostril of the trunk, which is kept in a lifted position, so that the solution is running up to the base of the trunk. The mixture of the solution and trunk mucus is collected in sterile plastic bags by active blowing of the elephant. Chemicals were all from Merck, Austria, unless otherwise stated. Restriction enzymes and kits for DNA extraction and purification were from New England Biolabs, USA. Oligonucleotides and synthetic genes were custom synthesised at Eurofins Genomics, Germany.

### Ethics declaration

We confirm that the trunk wash performed to provide a sample of the mucus was carried out as a routine procedure to monitor the health of elephants at the Vienna Zoo and in accordance with relevant guidelines and regulations.

### Trunk wash fractionation

Trunk wash was centrifuged for 1 h at 10,000 g, the supernatant was dialyzed against 50 mM Tris–HCl buffer, pH 7.4 and concentrated by ultrafiltration in the Amicon stirred cell, then fractionated by anion-exchange chromatography on HiPrep-Q 16/10 column, 20 mL (Bio-Rad), along with standard protocols.

### Protein alkylation and digestion, and mass spectrometry analysis

SDS-PAGE gel portions of proteins from whole elephant trunk wash (for component identification), chromatographic fractions of the elephant trunk wash (for PTMs analysis) or SDS-PAGE gel bands of LafrOBP1 expressed in *P. pastoris* were in parallel triturated, washed with water, *in gel*-reduced, S-alkylated, and digested with trypsin (Sigma, sequencing grade). Resulting peptide mixtures were desalted by μZip-TipC18 (Millipore) using 50% (v/v) acetonitrile, 5% (v/v) formic acid as eluent, vacuum-dried by SpeedVac (Thermo Fisher Scientific, USA), and then dissolved in 20 μL of aqueous 0.1% (v/v) formic acid for subsequent MS analyses by means of a nanoLC-ESI-Q-Orbitrap-MS/MS system, comprising an UltiMate 3000 HPLC RSLC nano-chromatographer (Thermo Fisher Scientific) interfaced with a Q-ExactivePlus mass spectrometer (Thermo Fisher Scientific) mounting a nano-Spray ion source (Thermo Fisher Scientific). Chromatographic separations were obtained on an Acclaim PepMap RSLC C18 column (150 mm × 75 μm ID; 2 μm particle size; 100 Å pore size, Thermo Fisher Scientific), eluting the peptide mixtures with a gradient of solvent B (19.92/80/0.08 v/v/v water/acetonitrile/formic acid) in solvent A (99.9/0.1 v/v water/formic acid), at a flow rate of 300 nL/min. In particular, solvent B started at 3%, increased linearly to 40% in 45 min, then achieved 80% in 5 min, remaining at this percentage for 4 min, and finally returned to 3% in 1 min. The mass spectrometer operated in data-dependent mode in positive polarity, carrying out a full MS1 scan in the range *m/z* 345–1350, at a nominal resolution of 70,000, followed by MS/MS scans of the 10 most abundant ions in high energy collisional dissociation (HCD) mode. Tandem mass spectra were acquired in a dynamic *m/z* range, with a nominal resolution of 17,500, a normalized collision energy of 28%, an automatic gain control target of 50,000, a maximum ion injection time of 110 ms, and an isolation window of 1.2 m*/z*. Dynamic exclusion was set to 20 s^36^.

### Bioinformatics for peptide identification and post-translational modification assignment

Raw mass data files were searched by Proteome Discoverer v. 2.4 package (Thermo Fisher Scientific), running the search engine Mascot v. 2.6.1 (Matrix Science, UK), Byonic™ v. 2.6.46 (Protein Metrics, USA) and Peaks Studio 8.0 (BSI, Waterloo, Ontario, Canada) software, both for peptide assignment/protein identification and for post-translational modification analysis.

In the first case, analyses were carried out against a customized database containing protein sequences downloaded from NCBI (https://www.ncbi.nlm.nih.gov/) for superorder Afrotheria (consisting of 192,838 protein sequences, December 2021) plus the most common protein contaminants and trypsin. Parameters for database searching were fixed carbamidomethylation at Cys, and variable oxidation at Met, deamidation at Asn/Gln, and pyroglutamate formation at Gln. Mass tolerance was set to ± 10 ppm for precursors and to ± 0.05 Da for MS/MS fragments. Proteolytic enzyme and maximum number of missed cleavages were set to trypsin and 3, respectively. All other parameters were kept at default values. In the latter case, raw mass data were analyzed against a customized database containing LafrOBP1 (XP_023395442.1) protein sequence plus the most common protein contaminants and trypsin, allowing to search Lys-acetylation (Δm =  + 42.01), Ser/Thr/Tyr-phosphorylation (Δm =  + 79.97), and the most common mammals N-linked glycans at Asn and O-linked glycans at Ser/Thr/Tyr, using the same parameters previously set. The max PTM sites per peptide was set to 2.

Proteome Discoverer peptide candidates were considered confidently identified only when the following criteria were satisfied: (i) protein and peptide false discovery rate (FDR) confidence: high; (ii) peptide Mascot score: > 30; (iii) peptide spectrum matches (PSMs): unambiguous; (iv) peptide rank (rank of the peptide match): 1; (v) Delta CN (normalized score difference between the selected PSM and the highest-scoring PSM for that spectrum): 0. Byonic peptide candidates were considered confidently identified only when the following criteria were satisfied: (i) PEP 2D and PEP 1D: < 1.0 × 10^−5^; (ii) FDR: 0; (iii) q-value 2D and q-value 1D: < 1.0 × 10^−5^. An FDR value of 1% was specified as the cut-off of false discovery rate for peptides identification by Peaks Studio. Manual interpretation and verification of the candidate spectra were always performed.

### Yeast expression and purification of proteins

The gene encoding OBP1 was cloned between XhoI and NotI restriction sites into the pPIC9 vector downstream of the α-factor secretion signal peptide. The following set of primers was used: 5’-AACTCGAG AAA AGA ATG CTG GAA GAA CC-3’; 5’-AAGCGGCCGC TTA GGT CAG TGT TTC CGG-3’ both for EmaxOBP1 and LafrOBP1. The construct was linearized with BglII and used to transform electrocompetent yeast cells (strain GS115) by electroporation (1.5 kV, 25 µF). Transformed cells were cultured on minimal dextrose (MD) agar plates (1.5% agar, 1.34% w/v yeast nitrogen base (YNB), 2% glucose, 4 × 10^–5^% biotin) for 48 h at 30 °C. Then, colonies were transferred onto minimal methanol (MM) agar plates (1.5% agar, 1.34% w/v yeast nitrogen base (YNB), 0.5% methanol, 4 × 10^–5^% biotin) and incubated at 30 ºC for further 48 h. Several colonies were selected from MM plates and screened for protein expression. First, colonies were grown in 10 mL of buffered minimal glycerol (BMGY) medium (1% w/v yeast extract, 2% w/v peptone, 1.34% w/v yeast nitrogen base with ammonium sulfate without amino acids (YNB), 4 × 10^–5^% biotin, 100 mM potassium phosphate, pH 6.0, 1% v/v glycerol) at 29 ºC, shaking at 240 rpm for 24 h. To induce protein expression, cells were harvested by centrifugation (15 min, 3,000 g, room temperature), re-suspended in 50 mL of buffered minimal methanol (BMM) medium (1.34% w/v YNB, 4 × 10^–5^% biotin, 100 mM potassium phosphate, pH 6.0, 1% v/v methanol) and incubated at 30 ºC for 72 h. To maintain protein expression, every 24 h methanol was added in amounts of 1.5% v/v. At the same intervals, samples of the supernatants were analyzed on SDS-PAGE for protein expression. On the basis of the results obtained, large-scale expression was performed under the above conditions for 72 h. The supernatant containing the recombinant OBP was centrifuged for 1 h at 10,000 g, dialyzed against 50 mM Tris–HCl buffer, pH 7.4, and concentrated by ultrafiltration in the Amicon stirred cell. Purification was performed by anion-exchange chromatography on HiPrep-Q 16/10, 20 mL (Bio-Rad) column, followed by elution from a Phenyl Sepharose column with the linear gradient of 0.6—0 M ammonium sulfate in 50 mM Tris–HCl, pH 7.4.

### Disulfide assignment

A sample of recombinant LafrOBP1 (20 μg) was dissolved in 0.1 M tetraethylammonium bicarbonate (TEAB), pH 6.5, containing 4 M guanidinium chloride, and then treated with iodoacetamide (0.5 M final concentration) for 30 min, in the dark. The protein was precipitated by the addition of 6 vol of cold acetone at −20 °C, overnight. After centrifugation at 12,000 rpm at 4 °C, for 20 min, supernatant was removed, and the recovered pellet was dried. Recovered protein was dissolved in 0.05 M TEAB, pH 6.5 (2 µg/μL final concentration), treated with trypsin (1:30 w/w enzyme/substrate) for 16 h, at 37 °C, and then with chymotrypsin (1:30 w/w enzyme/substrate) for 16 h, at 37 °C. Protein digest was desalted with ZipTip C18 (Millipore, USA) and directly analyzed with a UltiMate 3000 HPLC RSLC nano-chromatographer (Thermo Fisher Scientific) linked to a Q-ExactivePlus mass spectrometer (Thermo Fisher Scientific), as reported above.

Dedicated BioPharma Finder v. 4.0 (Thermo Fisher Scientific) and pLink v. 2.3.9 software were used for database searching of mass spectrometric data, enabling the specific function of disulfide-linked peptides attribution, and applying the additional settings reported above for Proteome Discoverer, Byonic and Peaks Studio examinations. A confidence score > 95 for BioPharma Finder and/or an E-value < 1.0^–10^ for pLink results were considered for a reliable identification of disulfide-bridged peptides. Candidate spectra were always verified by manual interpretation.

### Protein Model

A three-dimensional model of LafrOBP1 was obtained with the Swiss Model software^30–32^, using the allergen Bosd2 structure (PDB: 4WFU.1.A) as a template. The figure was generated with Chimera software^37^.

### Ligand-binding assays

Affinities of the fluorescent probe N-phenyl-1-naphthylamine (1-NPN) to EmaxOBP1 and LafrOBP1 (both expressed in yeast) were evaluated by titrating a 2 μM solution of each protein in 50 mM Tris-HCl, pH 7.4 with 1 mM 1-NPN in methanol to final concentrations of 2–16 μM. Dissociation constants of other ligands were measured by displacement of the fluorescent reporter 1-NPN from the complex. Accordingly, solutions of protein and 1-NPN, both at the concentration of 2 μM in 50 mM Tris-HCl, pH 7.4, were titrated with 1 mM methanol solutions of each ligand to final concentrations of 2–16 μM. Emission spectra were recorded on a PerkinElmer FL 6500 spectrofluorimeter in a right-angle configuration, at room temperature, with slits of 5 nm for both excitation and emission, using 1 cm path quartz cuvettes. The excitation wavelength was set at 337 nm and intensities were recorded in correspondence with the peak maximum, around 390 nm. Data for 1-NPN were processed with Prism software.

Dissociation constants of other ligands were calculated from the corresponding [IC]_50_ values (the. concentration of each ligand halving the initial value of fluorescence), using the equation: K_d_ = [IC]_50_/1 + [1-NPN]/K_NPN_, where [1-NPN] is the concentration of free 1-NPN and K_NPN_ the. dissociation constant of the complex OBP/1-NPN.

### Digestion with phosphatase and glycosidase

Fractions 27 and 29 from trunk wash and a sample of LafrOBP1 expressed in *P. pastoris* were treated in parallel with Lambda phosphatase, O-glycosidase and PNGase along with the protocols provided by the manufacturers. The digestion products were analysed on SDS-PAGE. The product after digestion of recombinant LafrOBP1 with phosphatase was assayed for binding activity to (*Z*)-7-dodecenyl acetate in competitive fluorescent binding experiments, as reported above**.**

## Supplementary Information

Supplementary Information 1.

Supplementary Information 2.

## Data Availability

The datasets generated and/or analysed during the current study are available as follows: the mass spectrometry-based proteomic data have been deposited to the ProteomeXchange Consortium via the PRIDE^38^ partner repository with the dataset identifier PXD036751. All other data are reported in the manuscript files and in Supplementary information.
