# Supplementary material for: An odorant-binding protein in the elephant's trunk is finely tuned to sex pheromone (Z)-7-dodecenyl acetate
Source: Sci Rep. 2022 Nov 21;12:19982. doi: 10.1038/s41598-022-24214-5 (PMC9678865; doi:10.1038/s41598-022-24214-5)
Supplement: Supplementary file 1 — Supplementary Information 1. [file 41598_2022_24214_MOESM1_ESM.pdf]

Zaremska et al.:

An odorant-binding protein in the elephant's trunk is finely tuned to sex pheromone (*Z*)-7-dodecenyl acetate

Supplementary files

Table S1. Identification details of the proteins detected in the trunk wash of the African elephant. (*see Excel file*)

Table S2. Assigned post-translationally modified peptides identified in the tryptic digest of the natural and recombinant LafrOBP1. Reported are the peptide sequence, the peptide numbering within the protein sequence, the experimental/theoretical mass value of the observed parent ion, and the modified amino acid residue. All cysteines were reduced and carboxyamidomethylated before trypsin digestion. Post-translational modifications are reported in parenthesis (with mass increment). LTW: *L. africana* trunk wash.

LTW fraction 26

Assigned modified peptides	Position	MH_2^{2+} (exp./theor.)	MH_3^{3+} (exp./theor.)	MH_4^{4+} (exp./theor.)	Modified amino acid	MH^+ (exp./theor.)
R.HIEC(+57.02)HPDGETLLVIFY(phosphorylated: +79.97)TK.E	44-61		751.3596/ 751.3538		Y59	2252.0632/ 2252.0457
Y.TK(acetylated: +42.01)ENGICQLYNK.Q asp.	60-71		503.9172/ 503.9174		K61	1509.7360/ 1509.7366
K.ENGICQLYNK(acetylated: +42.01)QGQR.I	62-75		583.9507/ 583.9498		K71	1749.8365/ 1749.8337
G.K(acetylated: +42.01)VDFSFIQQAK.D asp.	88-98	676.8598/ 676.8644			K88	1352.7118/ 1352.7209
R.EK(acetylated: +42.01)DISEENYQAFLEFAVENGIPK.E	124-146		904.7766/ 904.7732		K125	2712.3142/ 2712.3039
R.EKDISEENYQAFLEFAVENGIPK(acetylated: +42.01)ENIVK.V	124-151			824.6568/ 824.6651	K146	3295.6038/ 3295.6370
K.ENIVK(acetylated: +42.01)VIDTDTCPETPT	147-163	987.4742/ 987.4725			K151	1973.9406/ 1973.9372
K.VIDTDT(phosphorylated: +79.97)CPETPT	152-163	714.7810/ 714.7839			T157	1428.5542/ 1428.5600

LTW fraction 27

Assigned modified peptides	Position	MH_2^{2+} (exp./theor.)	MH_3^{3+} (exp./theor.)	MH_4^{4+} (exp./theor.)	Modified amino acid	MH^+ (exp./theor.)
R.HIEC(+57.02)HPDGETLLVIFY(phosphorylated: +79.97)TK.E	44-61		751.3605/ 751.3538		Y59	2252.0659/ 2252.0457
Y.TK(acetylated: +42.01)ENGICQLYNK.Q asp.	60-71		503.9171/ 503.9174		K61	1509.7357/ 1509.7366

K.ENGICQLYNK(acetylated: +42.01)QGQR.I	62-75	875.4219/ 875.4208			K71	1749.8360/ 1749.8337
R.IDENWYTTNYEGK(acetylated: +42.01)VDFSFIQQAK.D	76-98		946.7886/ 946.7804		K88	2838.3502/ 2838.3257
R.EK(acetylated: +42.01)DISEENYQAFLEFAVENGIPK.E	124-146		904.7805/ 904.7732		K125	2712.3259/ 2712.3039
R.EKDIS(O-Glycan HexNAc$_5$Hex$_5$: +1825.66)EENYQAFLEFAVENGIPK.E	124-146			1124.7535/ 1124.7440	S128	4495.9906/ 4495.9543
R.EKDISEENYQAFLEFAVENGIPK(acetylated: +42.01)ENIVK.V	124-151			824.6632/ 824.6651	K146	3295.6294/ 3295.6370
K.ENIVK(acetylated: +42.01)VIDTDTCPETPT	147-163	987.4735/ 987.4725			K151	1973.9392/ 1973.9372
K.VIDTDT(phosphorylated: +79.97)CPETPT	152-163	714.7816/ 714.7839			T157	1428.5554/ 1428.5600

LTW fraction 28

**Assigned modified peptides**	**Position**	**MH$_2^{2+}$ (exp./theor.)**	**MH$_3^{3+}$ (exp./theor.)**	**MH$_4^{4+}$ (exp./theor.)**	**Modified amino acid**	**MH$^+$ (exp./theor.)**
R.HIEC(+57.02)HPDGETLLVIFY(phosphorylated: +79.97)TK.E	44-61		751.3602/ 751.3538		Y59	2252.0650/ 2252.0457
Y.TK(acetylated: +42.01)ENGICQLYNK.Q asp.	60-71		503.9171/ 503.9174		K61	1509.7357/ 1509.7366
K.ENGICQLYNK(acetylated: +42.01)QGQR.I	62-75	875.4234/ 875.4208			K71	1749.8390/ 1749.8337
R.EK(acetylated: +42.01)DISEENYQAFLEFAVENGIPK.E	124-146		904.7733/ 904.7732		K125	2712.3043/ 2712.3039
R.EKDIS(O-Glycan HexNAc$_5$Hex$_5$: +1825.66)EENYQAFLEFAVENGIPK.E	124-146			1124.7520/ 1124.7440	S128	4495.9846/ 4495.9543
R.EKDISEENYQAFLEFAVENGIPK(acetylated: +42.01)ENIVK.V	124-151			824.6651/ 824.6651	K146	3295.6369/ 3295.6370
K.ENIVK(acetylated: +42.01)VIDTDTCPETPT	147-163		658.6500/ 658.6509		K151	1973.9344/ 1973.9372
K.VIDTDT(phosphorylated: +79.97)CPETPT	152-163	714.7842/ 714.7839			T157	1428.5606/ 1428.5600

LTW fraction 29

**Assigned modified peptides**	**Position**	**MH$_2^{2+}$ (exp./theor.)**	**MH$_3^{3+}$ (exp./theor.)**	**MH$_4^{4+}$ (exp./theor.)**	**Modified amino acid**	**MH$^+$ (exp./theor.)**
R.HIEC(+57.02)HPDGETLLVIFY(phosphorylated: +79.97)TK.E	44-61		751.3582/ 751.3538		Y59	2252.0590/ 2252.0457
Y.TK(acetylated: +42.01)ENGICQLYNK.Q asp.	60-71		503.9167/ 503.9174		K61	1509.7345/ 1509.7366
K.ENGICQLYNK(acetylated: +42.01)QGQR.I	62-75	875.4193/ 875.4208			K71	1749.8308/ 1749.8337

R.EK(acetylated: +42.01)DISEENYQAFLEFAVENGIPK.E	124-146		904.7739/ 904.7732		K125	2712.3061/ 2712.3039
R.EKDIS(O-Glycan $HexNAc_5Hex_5$: +1825.66)EENYQAFLEFAVENGIPK.E	124-146			1124.7527/ 1124.7440	S128	4495.9874/ 4495.9543
R.EKDISEENYQAFLEFAVENGIPK(acetylated: +42.01)ENIVK.V	124-151			824.6645/ 824.6651	K146	3295.6346/ 3295.6370
K.ENIVK(acetylated: +42.01)VIDTDTCPETPT	147-163	987.4764/ 987.4725			K151	1973.9450/ 1973.9372
K.VIDTDT(phosphorylated: +79.97)CPETPT	152-163	714.7844/ 714.7839			T157	1428.5610/ 1428.5600

LafrOBP1* from *P. pastoris

**Assigned modified peptides**	**Position**	**MH_2^{2+} (exp./theor.)**	**MH_3^{3+} (exp./theor.)**	**MH_4^{4+} (exp./theor.)**	**Modified amino acid**	**MH^+ (exp./theor.)**
R.HIEC(+57.02)HPDGETLLVIFY(phosphorylated: +79.97)TK.E	44-61		751.3597/ 751.3538		Y59	2252.0635/ 2252.0457
Y.TK(acetylated: +42.01)ENGICQLYNK.Q asp.	60-71		503.9169/ 503.9174		K61	1509.7351/ 1509.7366
K.ENGICQLY(phosphorylated: +79.97)NKQGQR.I	62-75	894.3963/ 894.3986			Y69	1787.7848/ 1787.7895
K.ENGICQLYNK(acetylated: +42.01)QGQR.I	62-75	875.4218/ 875.4208			K71	1749.8358/ 1749.8337
R.EK(acetylated: +42.01)DISEENYQAFLEFAVENGIPK.E	124-146	1356.6688/ 1356.6558			K125	2712.3298/ 2712.3039
R.EKDIS(O-Glycan $HexNAc_5Hex_5$: +1825.66)EENYQAFLEFAVENGIPK.E	124-146			1124.7517/ 1124.7440	S128	4495.9850/ 4495.9543
R.EKDISEENYQAFLEFAVENGIPK(acetylated: +42.01)ENIVK.V	124-151			824.6564/ 824.6651	K146	3295.6022/ 3295.6370
K.VIDTDT(phosphorylated: +79.97)CPETPT	152-163	714.7822/ 714.7839			T157	1428.5566/ 1428.5600

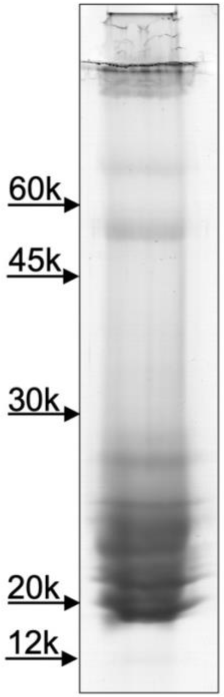

Figure S1. Gel electrophoresis analysis under denaturing conditions (SDS-PAGE) of a sample of the trunk wash from the African elephant. Before SDS-PAGE, the sample was centrifuged, dialyzed and concentrated.

```
                                                               P
                                                               P
DPLEEPLLDE YCLEISGTWY TIYEASANIE VISENSPLSG YFRHIECHPD GETLLVIFYT   60

A          A                 A
A       P  A
KENGICQLYN KQGQRIDENW YTTNYEGKVD FSFIQQAKDF LLIYVINKNE EGNVFEVVGA  120

    A  G                   A     A     P
    A  G                   A           P
LAREKDISEE NYQAFLEFAV ENGIPKENIV KVIDTDTCPE TPT                    163
```

Figure S2. Post-translational modifications in natural LafrOBP1 from the trunk wash of the African elephant (in green) and in recombinant protein expressed in *P. pastoris* (in blue). Modified amino acids are in bold. **P**: Phosphorylation, **A**: acetylation, **G**: O-glycosylation.

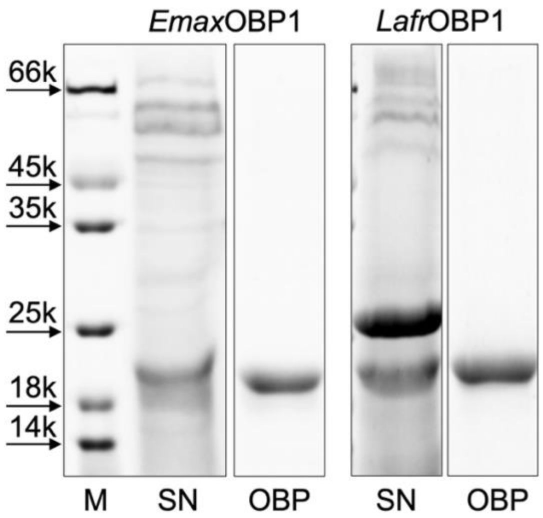

Figure S3. Expression of OBP1 from the Asian (Emax) and the African (Lafr) elephants in the yeast *Pichia pastoris*. **M**: molecular weight markers; **SN**: crude supernatant from the yeast culture; **OBP**: sample of purified protein.

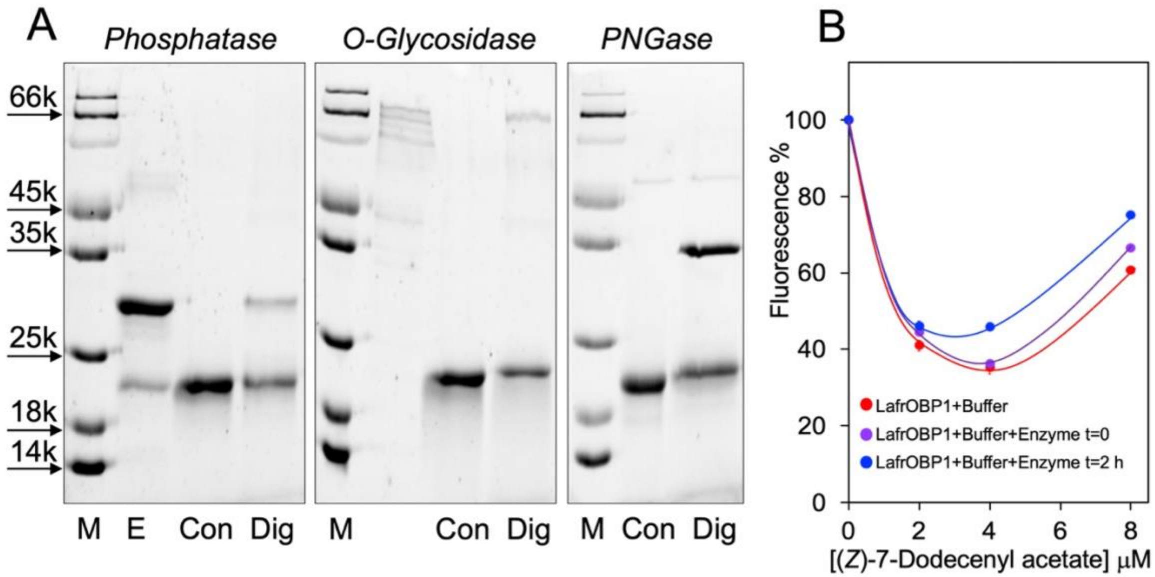

Figure S4. (**A**) Digestion of recombinant LafrOBP1 with phosphatase, O-glycosidase and PNGase. Treatment of the protein with any of the three enzymes did not modify its migration on SDS-PAGE. M: molecular weight markers; E: enzyme; Con: control before digestion; Dig: digested product. (**B**) Effect of digestion of recombinant LafrOBP1 with phosphatase on its binding activity towards the pheromone (*Z*)-7-dodecenyl acetate. The affinity is not affected after 2 h of digestion at 30 °C. The increase of the fluorescence at higher concentrations of ligand is due to formation of micelles by some amphiphilic ligands, which could entrap molecules of 1-NPN (Sun et al. 2012; Leal and Leal 2015; Pelosi, Zhu, et al. 2018).
